# Hormonal Regulation and Crosstalk of Auxin/Cytokinin Signaling Pathways in Potatoes In Vitro and in Relation to Vegetation or Tuberization Stages

**DOI:** 10.3390/ijms22158207

**Published:** 2021-07-30

**Authors:** Oksana O. Kolachevskaya, Yulia A. Myakushina, Irina A. Getman, Sergey N. Lomin, Igor V. Deyneko, Svetlana V. Deigraf, Georgy A. Romanov

**Affiliations:** Timiryazev Institute of Plant Physiology, Russian Academy of Sciences, Botanicheskaya 35, 127276 Moscow, Russia; vatrushbox@mail.ru (O.O.K.); yulia-myakushina@yandex.ru (Y.A.M.); getman_i@mail.ru (I.A.G.); losn1@yandex.ru (S.N.L.); igor.deyneko@inbox.ru (I.V.D.); sdeigraf@mail.ru (S.V.D.)

**Keywords:** auxin, cytokinin, phytohormone signaling, signaling crosstalk, sugar effects, gene expression, gene clustering, promoter *cis*-elements

## Abstract

Auxins and cytokinins create versatile regulatory network controlling virtually all aspects of plant growth and development. These hormonal systems act in close contact, synergistically or antagonistically, determining plant phenotype, resistance and productivity. However, the current knowledge about molecular interactions of these systems is still scarce. Our study with potato plants aimed at deciphering potential interactions between auxin and cytokinin signaling pathways at the level of respective gene expression. Potato plants grown on sterile medium with 1.5% (vegetation) or 5% (tuberization) sucrose were treated for 1 h with auxin or cytokinin. Effects of these two hormones on expression profiles of genes belonging to main signaling pathways of auxin and cytokinin were quantified by RT-qPCR. As a result, several signaling genes were found to respond to auxin and/or cytokinin by up- or down-regulation. The observed effects were largely organ-specific and depended on sucrose content. Auxin strongly reduced cytokinin perception apparatus while reciprocal cytokinin effect was ambiguous and sucrose-dependent. In many cases, functional clustering of genes of the same family was observed. Promoters in some clusters are enriched with canonic hormone-response *cis*-elements supporting their direct sensitivity to hormones. Collectively, our data shed new light on the crosstalk between auxin- and cytokinin signaling pathways.

## 1. Introduction

Auxins and cytokinins (CKs) are considered the most important plant hormones, responsible for fundamental traits of the plant organism [1,2,3]. These hormones determine the uniqueness of the plant hormonal system: the main sites of their synthesis are localized at opposite plant body poles, auxins at the top and CKs at the bottom. From their synthesis sites, these hormones move along the central plant axis in opposite directions. This pivotal auxin–CK countercurrent creates hormonal gradients that affect cell behavior and regulate proliferative growth [4,5,6,7]. Both hormones act synergistically in stimulating cell division but antagonistically in shoot or root branching [2,8,9,10,11]. In this way, these two hormones determine, to a large extent, the overall plant phenotype. Other important plant traits including organogenesis and resistance to biotic and abiotic stresses are also auxin-/CK-dependent [12,13,14,15,16,17,18,19]. In the 21st century, great progress has been achieved in dissecting the molecular mechanisms of auxin and CK perception and signal transduction [2,20,21,22,23,24,25]. Although totally different, both mechanisms turned out to be specific for plants; they do not copy molecular paths typical for animal hormones. The in planta crosstalk between auxin and CK at the various levels (molecular, cellular, interorganic) is now in the focus of studies by plant biologists [2,21,26,27,28,29,30]. However, little is known so far about the interaction of auxin and CK at the hormone signaling level. Furthermore, the available fragmentary information was obtained mainly for plants of one species—*Arabidopsis thaliana*. Evidently, other plants can have distinct features of species-specific interplay between auxin and CK signaling.

Potato is one of the most important crops; its tubers are extensively used worldwide for both nutritional and industrial purposes [31]. Plant hormones are known to participate in tuberization processes: auxins and CKs as promotive and gibberellins as suppressive factors [32,33,34]. Therefore, the molecular interplay between the phytohormones in potato is of special interest. A strong impetus to such studies has been given by the deciphering of the whole genome of the monoploid potato (‘Phureja’) some years ago [35]. Thereafter, potato genes related to auxin and CK regulatory systems were identified and some of encoded proteins were characterized [36,37,38,39,40]. The averaged expression profile of genes associated with the CK regulatory system in the diploid potato was recently demonstrated [39]. In the present work, we used the tetraploid commercial potato ‘Désirée’ to explore the effects of phytohormone treatments on the expression levels of the genes important for functioning of auxin- or CK-regulatory systems. In our previous studies, we have already noted the negative effect of ectopic endogenous auxin (IAA) on CK signaling [36]. Here, we have undertaken a systemic research of auxin and CK effects on signaling gene expression. In addition to phytohormones, the effect of sucrose on the expression of selected gene set was assessed, considering a strong impact of this sugar on potato development and tuber formation [41,42,43,44]. Multiple links between two signaling modules were uncovered, consistent with the occurrence of hormone response *cis*-elements in promoters of the signaling genes under study. The functioning of these regulatory links is largely plant status (sucrose content)- and organ-dependent.

## 2. Results and Discussion

### 2.1. Auxin Regulatory System

As for other hormones, the auxin signaling machinery is the central part of the whole auxin regulatory system. Targeted protein degradation mediates auxin signal transduction [24,25]. Three main proteins are involved in signaling: F-box TIR1/AFB-like receptors, Aux/IAA transcriptional repressors and ARF transcription factors (TFs). Aux/IAAs are short-lived multidomain proteins with affinity to both receptors and ARF TFs. At low auxin content, Aux/IAA repressors associate with ARFs and inhibit their activity. At higher auxin concentration, Aux/IAAs bind to F-box receptors which are components of so-called SCF-type E3 ubiquitin–protein ligase complexes. The latter label Aux/IAA repressors with ubiquitin, promoting their degradation by the 26S proteasome, therefore an eventual auxin effect upon signaling is a reduction of Aux/IAA repressors. Such a reduction leads to activation of ARF TFs which bind to promoters of the auxin-sensitive genes and regulate up or down their transcription, depending on the fine structure of ARF conserved regions [45,46].

Thus, the auxin signaling module TIR1/AFB–Aux/IAA–ARF consists of three types of tightly interacting proteins, and when studying the supposed effects on auxin signaling, this module should be considered mainly as a whole. In fact, the proteins involved in auxin signaling are very conserved among plant species. The sequenced genome of potato doubled monoploid ‘Phureja’ [35] encodes 5 StTIR1/AFB receptors (6 counterparts in Arabidopsis), 5 canonic StAux/IAA repressors (also 5 in Arabidopsis) and 14 canonic StARF transcription factors (12 in Arabidopsis) [37]. Here we used the tetraploid potato variety ‘Désirée’ growing in vitro to investigate the expression of genes encoding proteins constituting the above-mentioned auxin signaling module. The analysis of multiple gene expression at the transcriptional level was carried out for each of four main potato organs: leaves, stems, roots and tubers. In contrast to vegetative growth, for which 1.5% sucrose in the medium was sufficient, to stimulate tuberization, the plants were cultivated in parallel on a medium with 5% sucrose. The effects of both classical plant hormones known to positively affect potato tuberization: indol-3-acetic acid (IAA, auxin) and 6-benzyladenine (BA, cytokinin) were assessed (Table S1, Supplementary Data). The genes whose expression was not detected in any organ under any experimental condition were excluded from consideration. This exclusion mostly concerned several *StARF* genes which were silent at least in four organs studied.

#### 2.1.1. Auxin Signaling Genes in Potato and Regularities of Their Expression

**Figure 1:**
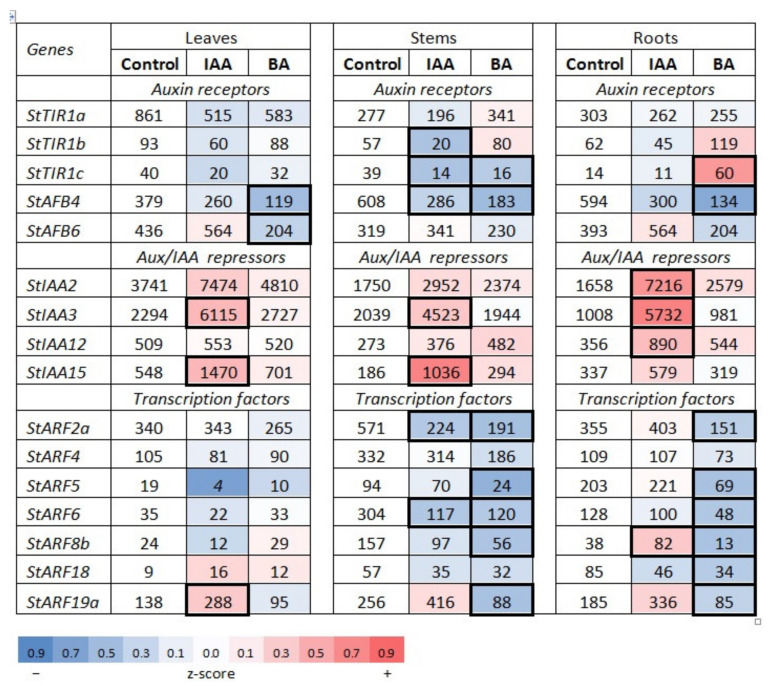
Effects of auxin (IAA) and cytokinin (BA) on the expression of the essential genes involved in auxin signaling in potato plants grown on medium containing 1.5% sucrose. Heatmap represents the logarithmic (lg) ratio of gene expression levels in potato plants, treated with IAA or BA, versus untreated (control) plants. Z-score of blue/red shows how strong is a decrease/increase of the expression level relative to control. Bold frames indicate cases of reliable differences (fold changes vs. control >2). Numbers in the cells represent absolute values of expression, averaged over two or more biological samples with three technical replicates each. Levels of gene expression too low (<10) to consider them as relevant are marked italics.

**Figure 2:**
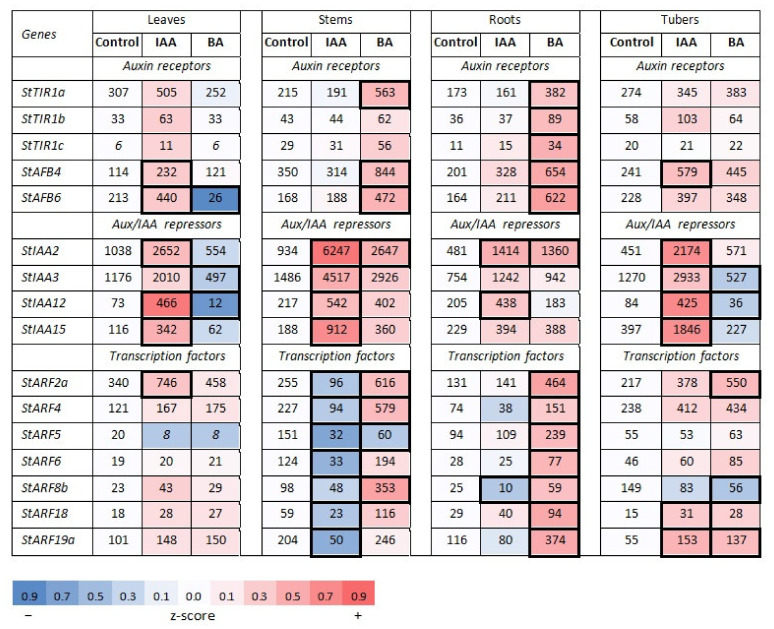
Effects of auxin (IAA) and cytokinin (BA) on the expression of the essential genes involved in auxin signaling in potato plants grown on medium with 5% sucrose. For heatmap details, see legend to Figure 1.

As in other plant genomes, in potato each type of auxin signaling genes is represented by a small gene family. For our study, the following genes were selected according to the expression criterion: *StTIR1a,b,c* and *StAFB4,6* as members of the auxin receptor gene family; *StIAA2,3,12,15* as canonic members of the *Aux/IAA* gene family, and *StARF2,4,5,6,8b,18,19a* as canonic members of the *ARF* gene family (Table S1). For each selected gene, specific primers were designed (Table S2). The character of gene expression in each family was obviously realized at different levels (Figure 1 and Figure 2). Among receptor genes, the expression of *StTIR1a*, *StAFB4* and *StAFB6* predominated irrespective of organ, sucrose content (1.5 or 5%) or hormone treatment. This clearly indicated the expression specificity at the level of the gene (+promoter) itself. As regards the organ specificity, some clear trends were also observed. In the leaves, the expression of *StTIR1a* predominated, while in stems and roots the transcription of *StAFB4* was most active. These trends were the same at both sucrose contents, though more pronounced at the lower one (Figure 1). In the tubers (5% sucrose), all three dominant receptor genes (*StTIR1a*, *StAFB4* and *StAFB6*) were expressed with similar intensities (Figure 2).

The averaged expression of *Aux/IAA* repressor genes was higher than that of receptor genes, aimed, evidently, at compensating the low stability of these proteins in the cell. Here also, the basic gene expression was unequal: the expression of *StIAA2* and *StIAA3* generally exceeded that of two other genes, *StIAA12* and *StIAA15*. As for the tubers, here *StIAA3* was dominating followed by *StIAA2* and *StIAA15* (Figure 1).

The genes encoding ARF transcription factors were usually expressed much less than *Aux/IAA* genes, suggesting higher stability of the former proteins. Among the whole set of seven genes under study, *StARF2a* and *StARF18* were, respectively, the leader and the outsider in the expression at both sucrose contents. It is also worth noting the much greater organ-dependent variability of *ARF* gene expression compared to receptor or *Aux/IAA* genes. For the latter, the expression profile of an individual gene across different organs is rather stable; virtually all organ-specific variations do not exceed 3.0–3.1-fold. By contrast, the organ-dependent variability in the expression of several *ARF* genes (*StARF5,6,8b,18*) can be much greater, reaching values in the range of 6–10-fold (Figure 1).

#### 2.1.2. Hormonal Effects on the Expression Profile of Auxin Signaling Genes

##### Effect of Auxin

Whole plants were treated in vitro with various phytohormones for 1 h (for details, see Section 3). Thereafter, the main plant organs were isolated, frozen and stored at −70 °C until RNA isolation. Individual gene expression in hormone-treated potatoes and mock-treated control plants was quantified by RT-qPCR with gene-specific primers (Table S2).

As was expected, the vast majority of samples did not show any significant difference between hormone-treated and control probes. Nevertheless, hormonal effects on gene expression profiles were indeed observed in some particular cases. Regarding auxin signaling genes, hormonal effects greatly depended on sucrose content in the medium, therefore data under conditions for vegetative growth (1.5% sucrose) or tuber formation (5% sucrose) are presented separately.

When plants were grown on medium with 1.5% sucrose, their treatment with auxin resulted in a moderate down-regulation of the expression of nearly all receptor genes except *StAFB6* whose activity did not decrease (Figure 1). By contrast, genes for Aux/IAA repressors commonly responded by up-regulation of their expression. Such a double effect on these gene families should lead to a shift in the repressor/receptor ratio in favor of the repressor counteracting against the excessive auxin dosage. Evidently, the auxin excess quickly switched on a negative feedback loop, seemingly in every potato organ. Although the ability of auxin to induce transcription of *Aux/IAA* genes was known long ago due to Arabidopsis studies [47,48], the parallel change in receptor gene expression reported here is novel and deserves in-depth study.

As for genes encoding ARF transcription factors, their response to auxin was not as uniform as the response of genes of other two signaling families. This difference is explained by the fact that ARFs are not as redundant as auxin receptors or Aux/IAA repressors. On the contrary, ARFs differ in their effects on gene transcription (may be positive or negative) and affect the expression of different sets of responsive genes. Nevertheless, most of *ARF* genes showed no significant changes in transcript numbers upon IAA treatment. This was especially true for *StARF4* stably expressing in each tested organ. By contrast, *StARF6* and *StARF19a* exhibited trends to decrease or increase their expression, respectively, in all auxin-treated organs. Other differently expressed genes (DEGs) of this family were transcribed too low, rendering any visible difference not enough relevant (Figure 1).

In potatoes cultivated on a medium with 5% sucrose (conditions for tuberization), the expression of genes for auxin receptors no longer decreased (Figure 2). On the contrary, a trend to an increase in their transcriptional activity can be noted, especially for the gene *StAFB4*, the expression of which rose more than 2-fold in leaves and tubers and 1.63-fold in roots. Collectively, the expression of all receptor genes in leaves almost doubled (DEG index 1.86, compared to DEG index 0.78 of the same genes in plants grown on a medium with 1.5% sucrose). Thus, the difference in the auxin effect on the expression of cognate receptor genes between plants grown on 1.5% or 5% sucrose seems to be significant, at least in leaves (DEG index ratio was 1.86/0.78 = 2.38).

By contrast, auxin promoted the expression of *Aux/IAA* repressor genes at both sucrose contents, indicating that their quick and positive response to hormone is due to the intrinsic properties of the genes themselves (Figure 2). The averaged DEG indexes of this gene family for all tested organs were rather close regardless the sucrose content (DEG indexes 2.65 and 3.14 for 1.5% or 5% sucrose, respectively), so we may conclude that one of the earliest and clear metabolic changes in potatoes treated with auxin is roughly three-fold increase in *Aux/IAA* gene expression. Some organ/sucrose specificity of the transcription induction can exist as well, for example, at 1.5% sucrose maximum DEG index was detected in roots and minimum in stems whereas at 5% this ratio was reversed. However, the question remains whether this sucrose-associated apparent organ specificity of *Aux/IAA* gene induction was caused by different physiological stages of plants (vegetative growing or tuber forming) or by different sucrose contents directly.

As was expected, most of the *ARF* TF genes changed their expression in response to auxin treatment less than two-fold, which is commonly considered as the absence of a relevant transcriptional effect. More pronounced changes of gene expression were organ-specific and not so uniform as in case of *Aux/IAA* genes. The only massive change under high sucrose condition, which may attract attention, was the expression decrease observed uniquely in stems (Figure 2). In this single organ, all (!) tested genes of the *ARF* family reduced their expression upon auxin treatment more than two-fold, averaged DEG index was 0.31 (more than three-fold difference). The physiological reason of such change is still unclear. Interestingly, similar trend though much less pronounced was manifested also by plants grown on medium with low sucrose (Figure 1). Here two *ARF* genes (*StARF2a* and *StARF6*) showed rather strong reduction in expression (DEG index 0.38–0.39), the expression of three more genes (*StARF5*, *StARF8* and *StARF18*) was tentatively reduced as well, though to less extent (DEG index 0.61–0.74).

##### Effect of Cytokinin

CKs are known to interplay tightly with auxins in regulation of the basic physiological processes; therefore, the crosstalk between these phytohormones at the level of molecular signaling is of special interest. When potato plants cultivated on medium with low (1.5%) sucrose were treated with cytokinin (BA), the hormonal effect on auxin receptor gene expression was rather limited and when occurred was mostly negative (Figure 1). Some major receptor genes (*StAFB4*, *StAFB6*) were down-regulated by BA in all or most organs tested. This decreasing trend was noted also for the expression of other receptors in particular organs (major receptor gene *StTIR1* in leaves and probably roots too; minor receptor gene *StTIR1c* in leaves and especially in stems). The decrease in receptor amount normally weakens the action of cognate hormone (namely auxin), but here other components of the entire signaling module, especially Aux/IAA repressors, can exert strong influence on the signaling output. In fact, BA-caused changes in expression of *Aux/IAA* genes are mostly ambiguous and can be viewed rather as trends and not as statistically significant alterations. The main trend seems to be a moderate (less than two-fold) increase in gene expression, characteristic for *StIAA2* (in all organs, DEG index 1.29–1.55) and *StIAA15* (in leaves and stems, DEG index 1.28 and 1.58, respectively) (Figure 1). As a result, CKs turned out to partly inhibit the signaling output by the auxin signaling module, evidently to less extent than auxin itself but anyway interfering with the molecular mechanism of auxin signaling.

Data on BA effects on *ARF* transcript contents were as a rule in agreement with the negative cytokinin impact on functioning of the auxin signaling pathway. When treated with BA, *ARF* genes either decreased their expression (this especially concerned stems and roots) or kept it unchanged (mainly in leaves), no significant raise in expression even of a single gene in a single organ was observed. The decrease in content of ARF TFs further weakens the potential of auxin to regulate the activity of responsive genes.

Therefore, unlike auxin effects on auxin signaling genes where intrinsic gene properties played a leading role in their expression, the CK effects on auxin signaling gene expression seemed to be more variable and mostly organ/sucrose-dependent.

### 2.2. Cytokinin Regulatory System

Similarly to auxin, the CK signaling machinery is composed of proteins belonging mainly to three families including that of CK receptors [22,23,49,50,51]. Additionally, the main targets of CK signaling are primary response genes which are distinct from those sensitive to auxin. However, the mechanisms of signal transduction for auxin and CK are different. Whereas auxin signaling is based on targeted protein degradation, CK signaling uses a classical two-component system (TCS); more precisely, its evolutionary advanced version termed multistep phosphorelay (MSP). Here, apart from membrane receptors—histidine kinases (HKs)—which become autophosphorylated upon hormone binding, there are also a small family of phosphotransfer proteins (HPts) shuttling between cytoplasm and nuclei, as well as their targets in nuclei, response regulator type B (RR-B) proteins which upon activation by “hot” phosphate can bind to the promoters of responsive genes and affect (usually activate) their transcription. This fundamental mechanism is strongly conserved among plant species.

The sequenced genome of potato ‘Phureja’ [35] encodes 3 StHK receptors (3 counterparts in Arabidopsis), 3 canonic phosphoransmitters (5 in Arabidopsis) and 7 canonic RR-B transcription factors (11 in Arabidopsis) [38,39]. Further, there are 8 canonic RR-type A (RR-A) proteins (10 in Arabidopsis) which are thought to interfere with RR-B in CK signal transduction. Similarly to *Aux/IAA* repressor genes which are also primary response genes to auxin, *RR-As* are primary response genes to CK creating negative feedback loop which prevents gene overexpression. Here, we investigated the expression profile of potato genes encoding proteins constituting the CK signaling pathway (Table S1). Potato plants in vitro were treated with BA, then cut into organs and frozen as in auxin signaling gene studies. All further procedures were performed similarly (see Section 3 and text above).

#### 2.2.1. Cytokinin Signaling Genes in Potato and Regularities of Their Expression

**Figure 3:**
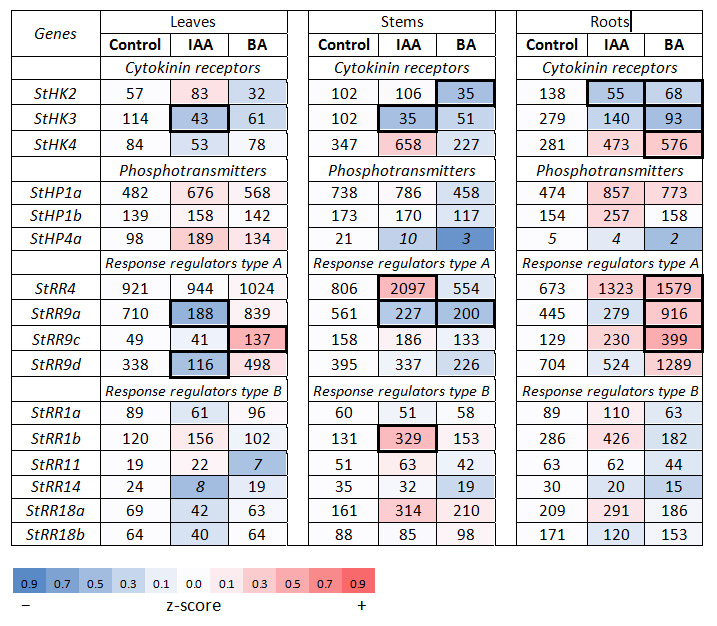
Effects of auxin (IAA) and cytokinin (BA) on the expression of the essential genes involved in cytokinin signaling in potato grown on medium with 1.5% sucrose. For heatmap details, see legend to Figure 1.

**Figure 4:**
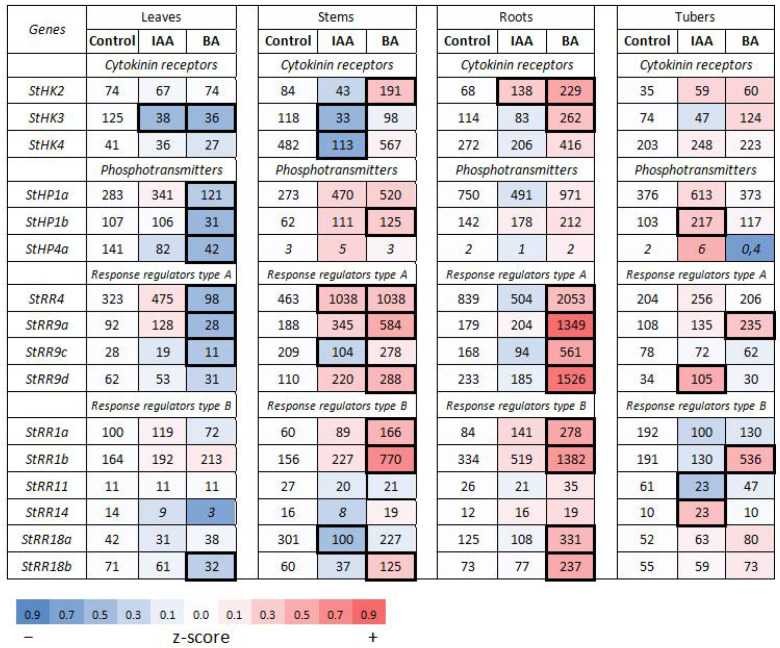
Effects of auxin (IAA) and cytokinin (BA) on the expression of the essential genes involved in cytokinin signaling in potato grown on medium with 5% sucrose. For heatmap details, see legend to Figure 1.

As in genomes of other plants, in potatoes each type of CK signaling genes is represented by a small gene family. For our study, all genes of the families encoding CK receptors (StHK2, 3, 4) and canonical phosphotransmitters (StHP1a, 1b, 4a) were used (Table S1). From the *StRR* genes, the set of *StRR4*, *9A*, *9C* and *9D* and the set of *StRR1a*, *1b*, *11*, *14*, *18a* and *18b* were selected as *RR-A* and *RR-B* genes, respectively; for each gene, specific primers were designed (Table S2). It is obvious that the specificity of gene expression in each family can be realized at different levels. Among receptor genes, *StHK4* expression prevailed in stems regardless of the sucrose content (1.5 or 5%) (Figure 3 and Figure 4). The expression of the same gene prevailed in tubers, treated or not with any hormone. The minimum of the *StHK4* activity was detected in leaves, similarly to Arabidopsis [52,53,54]. At the same time, the expression of *StHK3* was prevailing in leaves. This indicated the well-defined organ specificity of these two receptor gene expression. Thus, the main regularities revealed in the former study [38] were corroborated, at least qualitatively. Among *HP* genes, *StHP1a* expression was largely predominant in every organ and in every medium/treatment condition. This behavior of the gene clearly indicates that its high expression level is due primarily to the gene (+ promoter) itself. As for the *RR* genes, the expression of *StRR4* (type A *RR*) was far ahead of other type A and B genes, while there was no clear leader among the type B *RR* genes themselves. All of the aforementioned trends were similar for both ‘Désirée’ (Figure 3 and Figure 4) and DM potatoes [39], so they seem to be universal for any potato cultivar.

#### 2.2.2. Hormonal Effects on the Expression Profile of CK Signaling Genes

##### Effect of Cytokinin

The CK treatment effect on two families of CK signaling genes (encoding receptors or RR type A) in potato was mainly described in [38]. Here we extended our assay also on genes of two remaining signaling families: phosphotransmitters and RRs type B.

The effect of BA treatment on the expression of receptor genes at 1.5% sucrose was mainly neutral or negative; for *StHK2* and *StHK3*, the change was either significant (more than two-fold) or close to significant (Figure 3). The only clear positive effect occurred with the expression of *StHK4* gene in roots. Recall that in precedent study [38], *StHK4* was the only CK receptor gene positively responding to CK under low sucrose conditions. Genes encoding phosphotransfer proteins did not show any significant alteration due to BA treatment (changes in *StHP4a* expression were too low in absolute value to be considered as substantial). By contrast, *RR*-genes responded strongly and differently to CK administration: *RRs-A* were to a large extent up-regulated, especially in roots and to a lesser degree in leaves, while *RRs-B* were rather down-regulated, a trend most obvious in roots. Positive changes of *RR-A* gene expression were in accordance with BA-induced negative changes of the expression of other canonic signaling genes. As *RR-A* are negative regulators of cytokinin signaling, the enhancement of paralog gene expression complemented well the parallel decrease in *HK/HP/RR-B* gene expression. Altogether, these concerted changes can lead to a marked inhibition of CK signaling by excessive CKs, supporting the notion of the existence of the negative feedback regulation of CK signaling [55,56].

When plants were grown on tuberizing (5% sucrose) medium, the effect of BA on receptor/phosphotransmitter expression shifted from rather negative to rather positive. Under these conditions, a significant increase in expression of *StHK2* (stems, DEG index 2.28; roots, DEG index 3.35) and *StHK3* (roots, DEG index 2.29) receptor genes was observed (Figure 4). Main phosphotransmitter-encoding genes also showed a positive trend in their expression upon BA treatment: *StHP1a* in stems (DEG 1.91) and roots (DEG 1.30); and *StHP1b* in stems (DEG index 2.02) and roots (DEG index 1.49). All three expression leaders among *RR-B* genes—*StRR1a*, *StRR1b* and *StRR18a*—further increased their activity in roots and stems by 2.65–4.93-fold. All these results pointed to the enhancement of CK signaling by CK in potato, at least at 5% sucrose, in such organs as stems and roots. However, the role of RR-A regulators in the overall CK signaling should not be underestimated. Here strong BA-induced up-regulation of all *RR-A* genes was recorded, confined just to the same organs: stems (averaged DEG index 2.32) and roots (averaged DEG index 4.97) (Figure 4). So, in view of high degree of the rise in transcripts of *StRR-A* which are antagonistic to other components of the CK signaling module, the final effect of CK treatment of potatoes grown on medium with 5% sucrose, remains ambiguous.

##### Effect of Auxin

As was shown in various plant studies, auxin effect on CK regulatory system was rather negative, although the molecular details are not yet clear. In particular, in transgenic potato overproducing IAA the content of active CKs–nucleobases was reduced about two-fold [36]. However, what happens meanwhile at the level of signal transduction was unknown until very recently. To reveal genes belonging to CK signaling module and at the same time sensitive to auxin, potato plants were treated with auxin in a standard way. Isolated RNA was used to quantify individual gene expression versus mock-treated plants (control).

The general impression of the obtained results was consistent with the expected negative auxin effect, this time on genes involved in CK signaling. In particular, at 1.5% sucrose (vegetative growth) the expression of the *StHK3* receptor gene which had been shown to be dominant in leaves and co-dominant in roots [38] was reduced in all three organs tested (DEG index 0.44) (Figure 3). Genes for other signaling proteins, especially phosphotransmitters and remaining receptors, responded to auxin non-uniformly and mainly non-significantly (DEG index < 2); therefore, such effects should be viewed as implied trends rather than significant changes.

In the meantime, unique genes have been discovered that are associated with CK signaling and respond to auxin by rapidly increasing transcription. An example was *StRR4*, the dominant gene of the *RR-A* family, which was up-regulated 2.6-fold by auxin in stems and almost 2-fold in roots (Figure 3). Such an increase in *RR-A* family gene expression was even more pronounced in tuberizing plants grown on medium with 5% sucrose. Under these conditions *StRR4* was no longer alone to be activated by auxin (Figure 4). Other *RR-A* genes were significantly (DEG index > 2) up-regulated as well: *StRR9a* in stems and *StRR9d* in tubers. These changes in *RR-A* gene expression may be interpreted as a trend in the same direction (co-expression): *StRR4* was additionally up-regulated in leaves (DEG index 1.47) and roots (DEG index 1.26); *StRR9a* in leaves, stems and tubers (DEG indexes 1.39; 1.84 and 1.25, respectively). Recall that type A response regulators themselves are negative regulators of CK signaling, therefore auxin-induced activation of their expression obviously enhances the negative effect of decreasing receptor content.

Collectively, these concerted changes can be important to ensure the auxin-mediated decrease in CK signaling, especially in stems. It should be also noted that despite their reactivity to auxin, *StRR-A* remain genes of primary response to CK. This follows from the fact that in plants grown on tuberization medium these genes reacted to CK much more strongly (averaged DEG index 5.0) than to auxin (averaged DEG index 2.3) (Figure 4).

### 2.3. Clusterization of Gene Expression Changes and Effect of Sucrose

**Figure 5:**
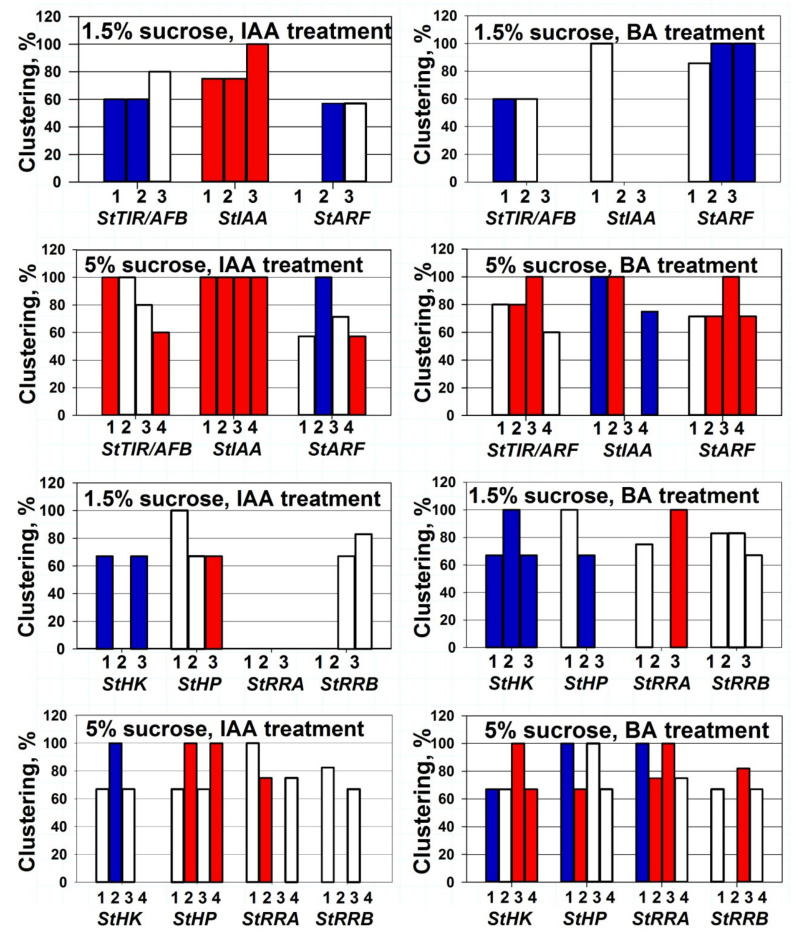
Cluster-like response (co-expression) of potato auxin/CK signaling genes to hormone treatments. 1, 2, 3, and 4 designate potato organs: leaves, stems, roots and tubers, respectively. Color indicates the change mode of cluster gene expression: up- or down-regulations are marked red or blue, respectively, and no significant changes (less than 1.5-fold) are not colored. Clustering % reflects percent of genes in the given family which respond uniformly to hormone treatment; clustering is considered relevant when it comprises more than 50% of genes of the given family.

The co-expression was long assumed to be a feature of paralog genes belonging to the same family, which means a uniform reaction to the defined stimulus [57,58]. We made an attempt to characterize at least qualitatively the extent of co-expression (functional clustering) of genes under study. The graphics in Figure 5 illustrate the apparent clustering of gene responses to hormonal treatments. When more than a half of the genes of a given family reacted to the stimulus more or less uniformly (co-expression), we considered these genes an apparent functional cluster. According to our rather mild clustering criteria, the majority of gene families under study responded to IAA or BA more or less uniformly as a whole cluster, but the extent of such clustering was rather variable. For example, CK signaling genes treated with IAA at 1.5% sucrose exhibited rather weak functional clustering: clusters formed only 7 variants (3 colorless bars) from 12, and among them, only one (colorless bar) cluster included 100% of family genes. In contrast, auxin signaling genes treated with IAA at 5% sucrose formed functional clusters at all 12 experimental variants (only 4 colorless bars), with seven 100% clusters (Figure 5). According to our estimation, the best co-expression demonstrated *StAux/IAA* genes up-regulated in all organs of potato treated with auxin. Much weaker, but still satisfactory, occurred co-expression of auxin receptor- and *StARF*-genes under the same conditions. Surprisingly, *StRR-A* genes, assumed, by analogy with other species, as primary response genes for CKs, did not form response clusters in some organs treated with BA. Moreover, in the leaves (5% sucrose), the direction of their co-expression was even reversed from positive to negative. The *StRR-B* genes represent another extreme case; almost all columns related to this family were colorless. This means that most of these genes did not markedly react to any hormonal treatment, so their bars in Figure 5 may be viewed as pseudoclustering. The same pseudoclustering was also characteristic of the CK receptor genes which, despite their functional redundancy, responded to hormones independently of each other. The opposite phenomenon, that is, the genuine co-expression of the studied genes in all 14 experimental assays of our study, seems to be a rare case. Among 16 auxin-related genes, only one pair—*StIAA3* and *15*—reacted always uniformly according to our criteria. Similarly, though a bit less synchronously (13 matches out of 14), *StTIR1a* and *StTIR1c* responded to hormones as a pair. As regarding genes belonging to the CK signaling module, here two genes encoding phosphotransfer proteins, StHP1a and 1b, responded almost uniformly (13 matches out of 14) to hormone treatments.

According to Figure 5, the apparent functional clustering of potato signaling genes was generally organ-specific. There were at least four cases when plants treated with the same hormone exhibited, depending on the organ, all three possible clustered reactions (positive, neutral or negative). In addition, sucrose exerted a strong influence on the gene expression changes provoked by hormone treatment. In some cases, sucrose was able to reverse even the very direction of changes in gene expression, not to mention its magnitude. For example, several gene clusters were down-regulated at low (1.5%) sucrose but up-regulated at 5% sucrose content upon treatment with the same hormone. These were auxin receptor genes in IAA-treated leaves, *StARF* TF genes in BA-treated stems and roots, *StRR-B* response regulator genes in BA-treated roots, and *StRR-A* response regulator and *StHP* phosphotransmitter genes in BA-treated stems (Figure 1, Figure 2, Figure 3, Figure 4 and Figure 5).

At the level of individual genes, higher sucrose enhanced hormonal effects resulting in increased number of significant DEG indexes as compared to plants grown at 1.5% sucrose. Interestingly, this sucrose effect depended also on gene family and used hormone. If, in the case of auxin signaling genes, high sucrose increased the effect of both hormones nearly equally (by 1.3–1.4-fold), in the case of CK signaling genes, treatment with auxin did not cause noticeable changes in the number of significant DEGs, but BA treatment at high sucrose tripled the significant DEG number (Figure 1, Figure 2, Figure 3 and Figure 4). The latter observation supports the idea of a close relationship between CK- and sugar signaling [11,59,60,61].

To assess the overall sucrose effect on auxin- and CK-signaling machineries, we compared the expression levels of the studied genes in control plants, grown on media with either 1.5% or 5% sucrose (Table S3). Results showed that a high sucrose markedly decreased the overall activities of both auxin and CK signaling modules. Suffice it to say that more than two-fold changes in expression were noted only downward (17 and 19 cases out of 48 for auxin and CK genes, respectively). The concerted drastic decrease in activity of all auxin receptor genes in leaves was especially impressive. At the same time, not a single case of a significant increase in the activity of any gene caused by sucrose was detected. A possible mechanism of this effect can also be associated with the similarity of the molecular action of CKs and sugars [11,59,60,61], in particular, with the suppression of auxin signaling by CKs noted earlier [36] and in this work. Auxins and CKs are well known to create and enhance sink strength of the respective organ. Therefore, the physiological meaning of the suppression of signaling by these two hormones seems obvious: to provide an advantage for the sink strength of the tuber over sink strengths of other potato organs. The latter must become donors and no longer acceptors upon the tuber emergence.

To conclude, the results presented in Figure 5 show that indeed, many redundant paralogous genes respond to hormonal stimulus with clusterized co-expression. However, the expectation of obligatory co-expression of the majority of paralogs belonging to the same gene clade [57,58] is not always justified. Sucrose exerts strong and variable effects on the responses of gene clusters and individual genes to hormone treatment. The promotive effect of sucrose on tuber growth is evidently associated with the decrease in the activity of sink-creating hormones (auxins and CKs) in non-tuber potato organs, thus ensuring the preferential sink strength of the tuber.

### 2.4. Promoter Analysis

The promoters of the genes under study were retrieved from the GenBank database where the ‘Phureja’ genome is uploaded. Not all promoters were available and some were sequenced only partly. Nevertheless, most full (about 1.5 kb upstream the transcription initiation site) promoters were obtained and analyzed by using corresponding software (see Section 3 for details).

**Figure 6:**
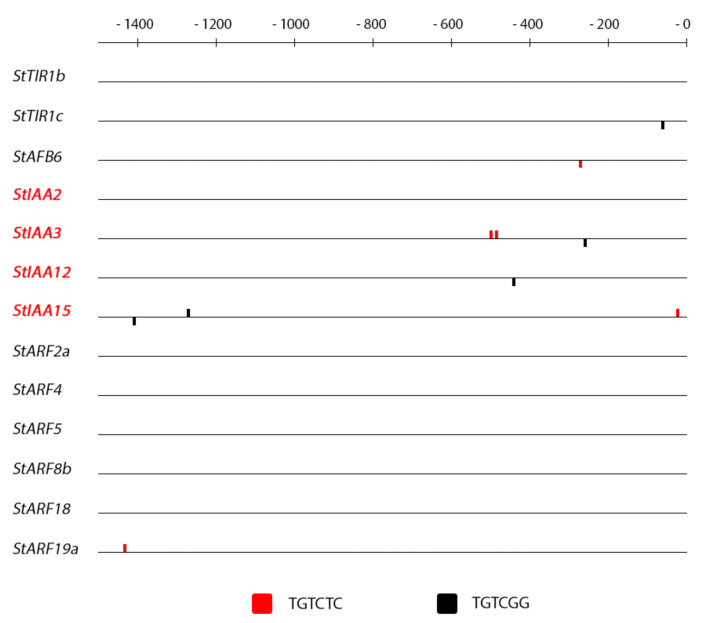
Pattern of auxin-specific *cis*-elements (AREs) in promoters of auxin signaling potato genes. Names of classical auxin-sensitive *Aux/IAA* genes are marked red. The scale on the top shows distance in bp upstream the transcription start.

**Figure 7:**
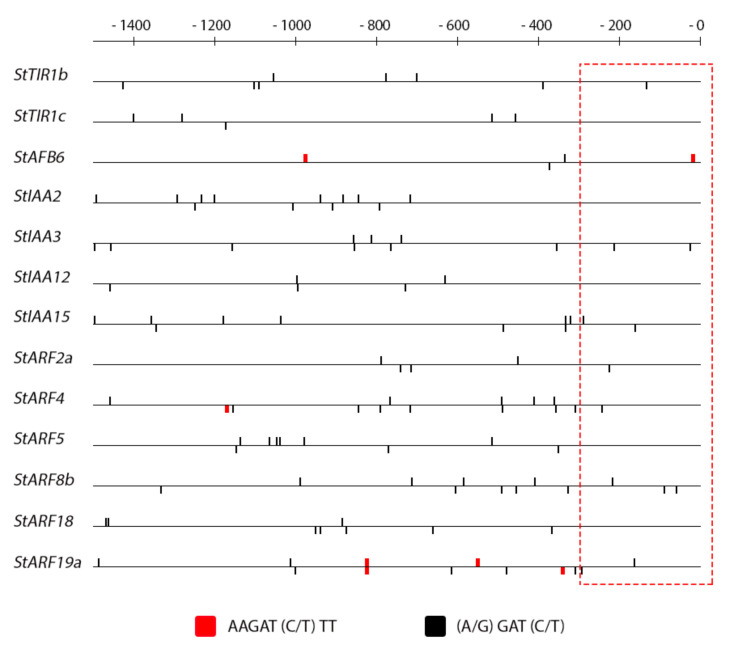
Pattern of CK-specific *cis*-elements (CREs) in promoters of auxin signaling potato genes. The scale on the top shows distance in bp upstream the transcription start. Presumable promoter zone rendering gene sensitive to CK is outlined by dotted red line.

**Figure 8:**
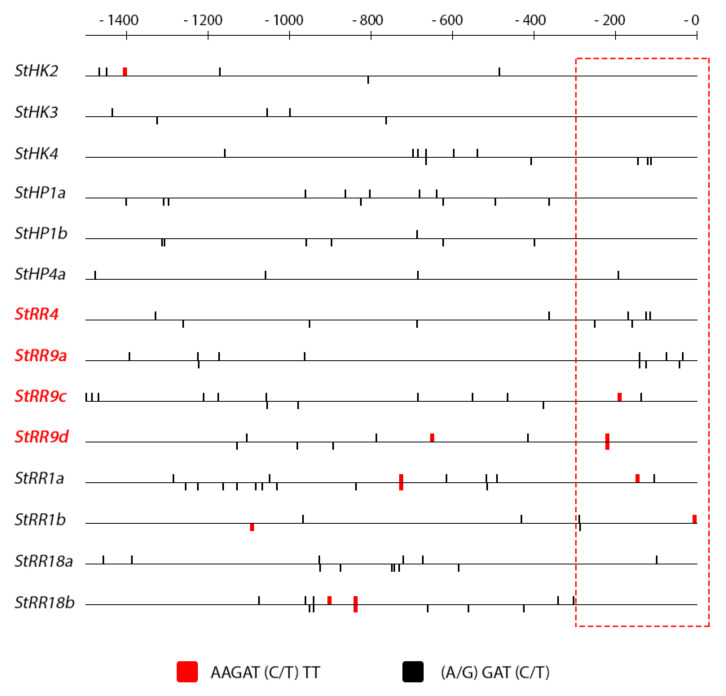
Pattern of CK-specific *cis*-elements (CREs) in promoters of CK signaling potato genes. Names of classical CK-sensitive *RR-A* genes are marked red. The scale on the top shows distance in bp upstream the transcription start. Presumable promoter zone rendering gene sensitive to CK is outlined by dotted red line.

**Figure 9:**
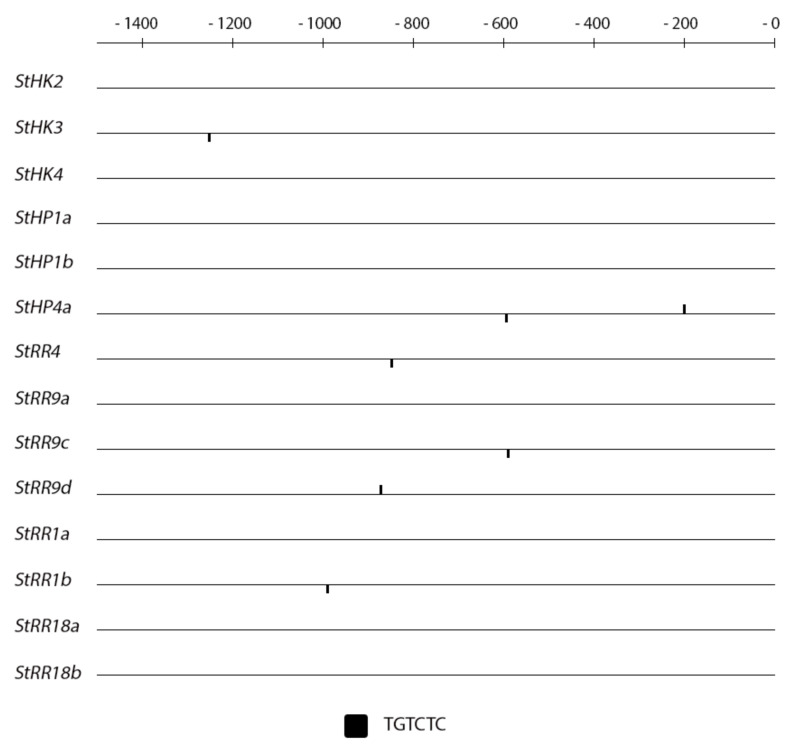
Pattern of auxin-specific *cis*-elements (AREs) in promoters of CK signaling potato genes. The scale on the top shows distance in bp upstream the transcription start. The position of ARE TGTCTC is shown; another ARE sequence TGTCGG is absent in these promoters.

Results of the analysis are demonstrated in Figure 6, Figure 7, Figure 8 and Figure 9, where universal consensus DNA loci (*cis*-elements) presumably ensuring the sensitivity of the promoter to auxin or CK are denoted. From the figures, clear difference in *cis*-element content between these two hormones is evident: the frequency of CK *cis*-elements is much higher than that of auxin *cis*-elements. This difference is obviously due to the fact that auxin-response elements (AREs) are longer and thus more specific than CK ones [(A/G)GAT(C/T)]. The occurrence of auxin *cis*-elements in promoters of potato genes belonging to different families was found to be non-chaotic. In the *Aux/IAA* family, almost every gene harbors at least one ARE in its promoter (Figure 6), the averaged amount of auxin *cis*-element per promoter of this gene cluster is equal to 1.75 (Table S4). Most of these AREs are localized within first 600 bp upstream transcription starting sites. Regarding other auxin-related gene families, ARE numbers per their promoter are much less frequent: 0.67 for receptor- and 0.17 for *ARF* gene clusters. These results strongly correlate with the relative sensitivity of these gene families to auxin treatment (Figure 1 and Figure 2): *StAux/IAA* family includes genes of primary response to auxin (integral DEG index for the sum of all samples was 2.84) while genes from other two families hardly reacted to auxin (analogous normalized DEG indexes were 1.04 and 0.99 for receptor- and *ARF* genes, respectively). The correlation coefficient between ARE numbers and DEGs values (including CK-related gene families) is as high as 0.93 (Table S4). Nevertheless genes encoding auxin receptors *StTIR1c* and *StAFB6* as well as *StARF19a* TF were found to contain at least one ARE in their promoters, suggesting the potential sensitivity of these genes to auxin as well. This potential sensitivity was evidently realized in our experiments, where these genes showed at least one significant response to auxin treatment (Figure 1 and Figure 2).

CK-response elements (CREs) are much more numerous than auxin ones and are present as multiple consensus sites in promoters of all studied genes regardless their classification (Figure 7). Since only minor part of the genes exhibited noticeable sensitivity to CK, obviously a great part of the identified short *cis*-elements are nonfunctional. Thus, the question of identification of a few functional elements among the predominant amount of non-functional ones still remains to be addressed.

In our previous work, a cluster of potato genes quickly responding to CK treatment has been revealed [38]. As in other plant species including Arabidopsis, these genes belong to the *RR* type A family, classical CK primary response genes [62,63]. In promoters of these potato genes, a specific patterning of CREs was demonstrated with a clear tendency to be closely spaced within approximately the first 300 bp upstream of the transcription start site. The present data are consistent with this inference since in other gene clusters these promoter areas close to transcription start are markedly depleted of CREs compared to *StRR*-*A* promoters (Figure 8 and Figure 9). CRE quantification in these promoter parts resulted in the averaged CRE numbers per promoter of 1.0, 0.33, 1.5 and 3.75 in *StHK*, *StHP*, *StRR-B* and *StRR-A* gene families, respectively. Note a clear advantage of *StRR-A* genes in this parameter. According to expression profiling (Figure 3 and Figure 4), normalized integral DEG indexes for the respective gene families were 1.10, 1.08, 1.61 and 1.76, resulting in rather high—0.87—correlation coefficient between two data rows (including data corresponding to auxin gene families, see Table S4). Regarding the auxin signaling genes, at least two of them are distinguished by the rich CRE content in their promoters (Figure 7): in the promoter of *StARF8b*, three short CREs are present within the proximal 300 bp zone, and in the promoter of *StARF19a*, there are two short CREs in the 300 bp zone followed by numerous CREs including four long ones. Both genes showed, in fact, rather high sensitivity to CK: *StARF8b* and *StARF19a* demonstrated five and four significant responses to CK treatment (out of seven assays in total), respectively (Figure 1 and Figure 2).

On the other hand, the presence of AREs in promoters of some CK signaling genes (Figure 9) renders these genes more sensitive to auxin treatment. The promoters of 6 CK signaling genes from a total of 14 harbor at least one ARE. When potato plants grown on medium with 1.5% sucrose were treated with IAA, more than 83% of genes with AREs in their promoters (*StHK3*, *StHP4*a, *StRR4*, *StRR9c*, *StRR9d*, *StRR1b*) responded to hormone with at least one significant change in gene expression profile (DEG index 2 and more). From the remaining eight genes lacking AREs, only two (25%) significantly changed their expression. A similar difference was observed when plants were grown on a tuberization medium with 5% sucrose. Thus, the occurrence of ARE(s) in promoters of CK signaling genes points to very plausible link between auxin- and CK signaling modules, implemented by these genes. The preferable occurrence of consensus *cis*-elements in promoters of hormone-sensitive gene clusters and high correlation coefficients between DEG values and response element numbers in proximal parts of promoters evidenced for the leading role of canonic regulatory *cis*-elements which determine the hormone-sensitivity of promoters of potato genes, similarly to other known plants. Of course, this does not negate the possibility that potato has additional species-specific *cis*-regulatory motifs. This suggestion is supported by the fact that the gene *StIAA2*, strongly up-regulated by auxin (Figure 1 and Figure 2), has no canonic AREs in its promoter.

As another explanation of this inconsistency, some differences can be anticipated in DNA sequences between the promoters of the full-sequenced potato ‘Phureja’ and slightly sequenced potato ‘Désirée’ genomes. In particular, *StIAA2* promoter in ‘Désirée’ may have ARE(s) which is (are) absent in orthologous promoter of ‘Phureja’. To assess the likelihood of the latter scenario, we compared the occurrence and positions of ARE and CRE *cis*-elements in promoters of CK receptor gene orthologs of ‘Phureja’ and ‘Désirée’ (recently uploaded to GenBank). Data showed (Figure S1) that the orthologous promoters of these two potato cultivars usually share identical patterns of functional *cis*-elements. However, some changes in promoter sequences of ‘Désirée’ genes were nevertheless detected. In one case an auxin-response *cis*-element (TGTCTC) arose in the promoter of one of the orthologs of the *StHK4* gene which in ‘Phureja lacks ARE. Such a mutation could render this *StHK4* paralog in ‘Désirée’ sensitive to auxin. Notably, any scenario with hormone-responsive *cis*-elements in promoters of signaling genes points to a direct effect of TFs of the signaling module of one hormone on the expression of genes of the signaling module of another hormone, and vice versa. The interaction of TFs (ARFs, RRs) with cognate promoter-located *cis*-elements can be rather complex, with the participation of other, including organ-specific, chromatin proteins [46].

### 2.5. Molecular Details of Crosstalk between Auxin- and CK Signaling Modules in Potatoes

The obtained data shed new light on the interaction mode between auxin- and CK signaling modules, exemplified by those of potato. Early auxin and CK effects were recorded 1 h after hormone treatment of plants grown in vitro. Two versions of growth media were employed, with low (1.5%) or high (5%) sucrose content, favorable for vegetative growth or tuber formation, respectively.

The revealed rapid changes in transcript content evidenced for the potential of these two phytohormones to impact reciprocally signaling outputs. More precisely, CK treatment of potato plants grown on medium with low (1.5%) sucrose led to a marked reduction of auxin signaling. This decrease concerned each organ under study: leaf, stem and root. At the molecular level, the expression of genes responsible for auxin perception was reduced up to 0.57–0.65 of the control values; mainly the receptor genes *StAFB4* and to less extent *StTIR1a* were implicated. In addition, the expression of *ARF* genes was reduced up to 0.39–0.80 of the control values. Among the last gene family, the expression of *StARF2a* was especially affected. Surprisingly, the expression of *StAux/IAA* repressor genes, contrary to expectations, was not down- but rather up-regulated by CK. This particular effect observed in all vegetative potato organs further contributed to the decrease in auxin signaling output.

Unlike potatoes grown at 1.5% sucrose, plants grown on medium with 5% sucrose reacted to CK treatment in a different way. In the aerial part of plants (leaves, stems), the final effect of CK treatment was ambiguous; the down-regulation of the receptor genes was found to occur only in leaves. In the other three organs CK clearly enhanced transcription of the receptor genes, especially *StAFB6* and *StAFB4*. Genes for ARF transcription factors were also induced by CK; *StARF2a* was particularly strongly affected.

Generally, according to amounts of implicated genes, the relationship between both modules was unequal: genes belonging to auxin system responded to CK treatment 2–3 times more often compared to reciprocal response of CK system genes to exogenous auxin administration. This imbalance resulted in a stronger effect of exogenous CK on auxin signaling module than vice versa. Globally, at 1.5% sucrose, exogenous CK reduced auxin signaling in every potato organ, i.e., in the whole plant. However, at 5% sucrose, the mode of CK action on auxin signaling radically changed from negative to positive, especially in stems and roots. In addition, the self-activation of CK signaling in these organs was detected as well. In turn, IAA treatment should strongly suppress CK signaling in stems at 5% sucrose: the expression of all three CK receptor genes as well as active gene for StRR18a TF were eventually down-regulated, while the expression of *RR-A* genes for negative regulators of CK signaling was, on the contrary, mostly up-regulated.

Data from the literature, though still fragmentary, are consistent with revealed regularities of auxin-CK signaling crosstalk. In Arabidopsis, auxin was shown to up-regulate genes encoding type A response regulators ARR7 and ARR15 in root embryonic cells [64] as well as pseudo phosphotransmitter AHP6 [65]. The anticipated auxin-mediated increase in the content of these negative regulators should markedly suppress the CK signaling output. Interestingly, auxin was reported to modulate the expression of the same genes—*ARR7* and *ARR15*—also in the shoot apical meristem (SAM), through the TF ARF5. Unlike the root effect, in the SAM auxin did not enhance, but, on the contrary, reduced the expression of these *RR-A* genes, rendering the latter organ more sensitive to CKs [56,66]. These regulatory links have a clear analogy in potato where *RR-A* genes were strongly down-regulated by IAA in the aerial organs (Figure 3). On the other side, CK was reported to affect the expression of auxin signaling genes as well. Type B response regulator ARR12 was reported to positively regulate the auxin signaling gene *ARF19* in root apical meristem (RAM) [67]. In another study, type B TFs ARR1 and ARR12 were shown to up-regulate the transcription of *Aux/IAA3* (*SHY2*) gene in root tips, depicting the possible pathway for RAM size regulation by CKs [68]. Notably, in potato too, one of *Aux/IAA* genes (*StIAA2*) was significantly up-regulated by BA (Figure 2). In most of the abovementioned cases, regulatory events are thought to occur directly, due to ARF or RR-B binding to hormone-response *cis*-elements in promoters of targeted genes. Some other possible links between auxin and CK signaling machineries, though considered yet preliminary, can be found in [69,70,71].

**Figure 10:**
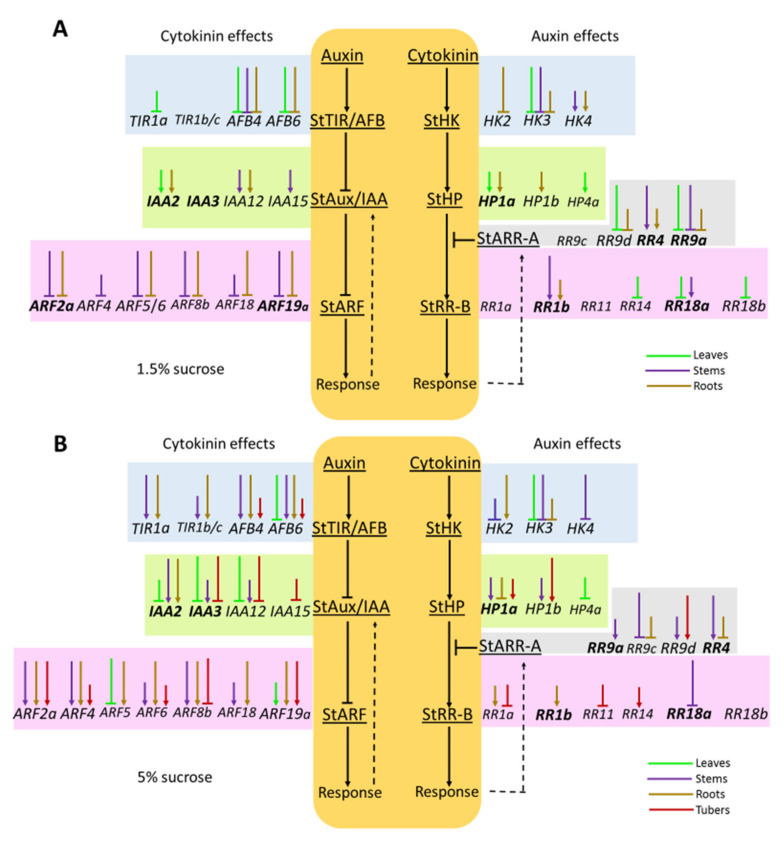
The scheme summarizing all putative links which can be implicated in auxin-CK crosstalk in potato at the signaling level. The schematic auxin- (on the left) and CK (on the right) signaling pathways reside in the center, and genes encoding defined protein family are grouped in a row at the cognate protein level. Colored arrows show potential interactions: pointed or blunted arrows indicate activation or inhibition, respectively. Arrows corresponding to each potato organ are marked with a distinct color. Long arrows indicate pronounced effects (fold changes >2), short arrows indicate apparent trends (fold changes >1.5 but <2). Data on plants grown on media with 1.5% (**A**) sucrose or 5% (**B**) sucrose are given separately.

In this context, our study was apparently the first systemic analysis of auxin and CK signaling genes which may be involved in direct crosstalk between these two signaling pathways. An essential outcome of the research is summarized in Figure 10, which demonstrates the main putative regulatory network connecting these signaling modules. These schemes can serve as a framework for forthcoming detailed investigations of the interplay between signaling modules of various plant hormones. We may envisage that late hormonal effects would be even more intense due to the fact that CK-affected late genes were much more abundant than early response genes, at least in Arabidopsis [63].

The apparent abundance of arrows in Figure 10 should not be misleading, since the diagram contains an abundance of all possible variants for the interaction of genes of the studied signaling systems. In fact, in each individual organ, and even more so in an individual cell, only a minor part of the indicated interactions can be effectively realized. Detailed studies of signaling crosstalks in individual organs, tissues and cells can be a topic of further in-depth studies in this area. On the other hand, recall that land plants evolved from charophycean algae about 500 million years ago [72]. They also inherited, from algae auxins and cytokinins, the most ancient signaling molecules [73]. Once the plant ancestors occupied the land, the auxin and cytokinin signaling systems underwent joint evolution. It would be strange if, over such a long period of tight co-evolution, the signaling modules of these ubiquitous hormonal systems did not form numerous regulatory interconnections. Another argument in favor of the multiplicity of links between signaling modules in the studied plants follows from the results of the promoter analysis (Figure S1). This analysis demonstrated that paralog genes of the tetraploid potato ‘Desiree’ may have in their promoters additional *cis*-regulatory elements absent in the basic monoploid ‘Phureja’ genome. Such extra *cis*-elements may obviously create additional regulatory interconnections. Thus, with an increase in ploidy degree, the number of potential regulatory links between different signaling systems can markedly increase. Short arrows denoting DEG trends may be evidently neglected as being formally non-significant. However, such trends may be indicators of developing late reactions or of distinct response to the stimulus of some of the genes-paralogs.

## 3. Materials and Methods

### 3.1. Plant Growth Conditions

The study was performed with potato plantlets (*Solanum tuberosum* L.) cv. ‘Désirée’ belonging to the mid-maturity group (http://www.europotato.org/) (accessed on 28 July 2021). Plants were propagated by single-node stem cuttings and grown in vitro at 20 °C and 16 h photoperiod on liquid Murashige-Skoog (MS) medium supplemented with 1.5% or 5% sucrose as described [38,42]. Visibly uniform plants grown for 5 weeks were used in vegetation and tuberization experiments. Plants cultivated in glass tubes in vitro were treated with different phytohormones (10^−6^ M): indole-3-acetic acid (auxin, IAA) or 6-benzyladenine (cytokinin, BA), dissolved in liquid medium. Tubes were inverted several times to assure uniform plant wetting with hormone solution and incubated for 1 h under standard conditions. Control plants were treated similarly with fresh liquid medium but without hormones. Finally, the plant organs (leaves and stems from the middle part of shoots, entire roots and fully formed microtubers 50–150 mg FW) were isolated, frozen in liquid nitrogen and kept at −70 °C until RNA isolation. All experiments were carried out in duplicate or triplicate, with 15 plants per replicate.

### 3.2. Quantitative Real-Time PCR

Total RNA was extracted from 100–250 mg of fresh tissues by means of TRIzol method (Invitrogen) and treated with RNAse-free DNase I (Evrogen, Moscow, Russia; 1 U/µL). cDNA was synthesized on the RNA template with MMLV reverse transcriptase (Evrogen) according to manufacturer’s protocol. The absence of genomic DNA in cDNA samples was confirmed by PCR with primers to intron-containing fragment of patatin gene. Quantitative gene expression was determined by quantitative Real time PCR (RT-qPCR) (Table S1) using ANK48 analyzer (Syntol, Moscow, Russia). DNA was amplified with qPCRmix-HS SYBR (Evrogen, Moscow, Russia); primers are listed in the Table S2. Gene-specific primers were designed by means of the Primer-Blast program (https://www.ncbi.nlm.nih.gov/tools/primer-blast/) (accessed on 28 July 2021) ensuring primer uniqueness; the best primer pairs were selected using the OligoAnalyzer tool (https://eu.idtdna.com/) (accessed on 28 July 2021). When possible, primers crossing the exon/intron boundary were chosen. Primer quality was additionally validated by melting amplicons generated by RT-qPCR as well as by amplicon size and purity determination using electrophoresis in 1% agarose gel. The conditions for RT-qPCR were as follows: pre-denaturation at 95 °C for 60 s, followed by 35 cycles of denaturation at 95 °C for 30 s, annealing at 60 °C for 15 s and a final extension step at 72 °C for 15 s. DNA sequences encoding potato elongation factor EF1a (GenBank acc. No AF126551) and cyclophilin CYC (GenBank acc. No AF126551) were employed (with similar results) as reference genes [74]. Each datum on transcript content used to calculate average values in Figure 1, Figure 2, Figure 3 and Figure 4 represents the mean value of three technical replicates.

### 3.3. Functional Clusters Determination

The gene expression response to hormonal stimuli was divided into three options: increased expression, weakened expression and no reaction. The criterion for the increase or decrease in expression was a change in the DEG index by more than 50% of the control level. Accordingly, the criterion for no response was no or only slight change in expression, not exceeding 50% of the control level. After this gene sorting, we determined functional clusters, the genes of which were characterized by the same type of response (co-expression) to hormones and accounted for more than half of the total number of studied genes of this family. Such co-expressed genes were considered to constitute an apparent functional cluster in some organ under defined conditions. As a control, the probability of random clustering (pseudo clusters) was assessed, which depends on the number of genes in the gene clade and reaches high values with a small (3–4) number of genes. If the actual clustering of genes did not clearly exceed the calculated probability of random clustering, the genes were considered not satisfying the functional clustering criterion.

### 3.4. Promoter Analysis

The nucleotide sequences of promoters of CK- and auxin signaling genes of potato *Solanum tuberosum* cv ‘Phureja’ were retrieved from GenBank (NCBI). For the analysis, we took promoter sequences of 1500 bp length from the transcription start. CK-sensitive *cis*-regulatory elements were for: long AAGAT(C/T)TT [75,76] and short (A/G)GAT(C/T) sequences [77,78,79]. Additionally, auxin-sensitive *cis*-regulatory elements (AREs), TGTCTC [80] and TGTCGG [45], were identified. A schematic representation of regulatory *cis*-element occurrence within promoter regions was performed using the TOUCAN 2 program [81].

### 3.5. Statistics

All data of transcript content represent the mean values (±SD) of at least two biological experiments with three technical replicates each. Raw values measured by RT-qPCR (Supplementary Data) were first normalized to the reference genes. Resulting values were used to calculate relative expression change between treated and control samples using lg (treated/control) [82]. The lg-ratios were used to draw heatmaps in Figure 1, Figure 2, Figure 3 and Figure 4. All calculations were performed in R (https://www.R-project.org/) (accessed on 26 February 2021). Gene expression in control plants and plants treated with auxin/cytokinin was compared using ANOVA. Pair-wise comparisons (control/treated) were performed using *t*-test for each hormone independently.

## 4. Conclusions

In the present study, we explored the effects of the most important plant hormones, auxins and CKs, on the functioning of the signaling machineries for the same hormones. As a model plant, the tetraploid potato cv. ‘Désirée’ grown in vitro was used. A number of genes belonging to auxin-signaling pathway were found to be regulated by CK, and vice versa. Taken together, the results revealed that, indeed, auxin and CK reciprocally influenced the signaling output of the respective pathways. These hormonal effects were mainly organ-specific and, as a rule, largely depended on sucrose content in the medium. Particularly, auxin acted mainly as a negative regulator of CK perception at all sucrose contents in media; by contrast, CK antagonized auxin signaling only at a low sucrose level, whereas at a high sucrose level CK acted differently, positively regulating the auxin perception. Taken together, our results point to the functioning of multiple molecular links between auxin- and CK signaling modules, justified by the occurrence of numerous response *cis*-elements for auxin and CK in promoters of the majority of respective signaling genes. This predicted crosstalk between auxin- and CK signaling pathways should be taken into account when considering the molecular aspects of the activity of these phytohormones.

## Data Availability

Not applicable.

## Supplementary Materials

The following are available online at https://www.mdpi.com/article/10.3390/ijms22158207/s1.
