# Supplementary material for: Hormonal Regulation and Crosstalk of Auxin/Cytokinin Signaling Pathways in Potatoes In Vitro and in Relation to Vegetation or Tuberization Stages
Source: Int J Mol Sci. 2021 Jul 30;22(15):8207. doi: 10.3390/ijms22158207 (PMC8347663; doi:10.3390/ijms22158207)
Supplement: Supplementary file 1 [file ijms-22-08207-s001.zip › Figure S1.pdf]

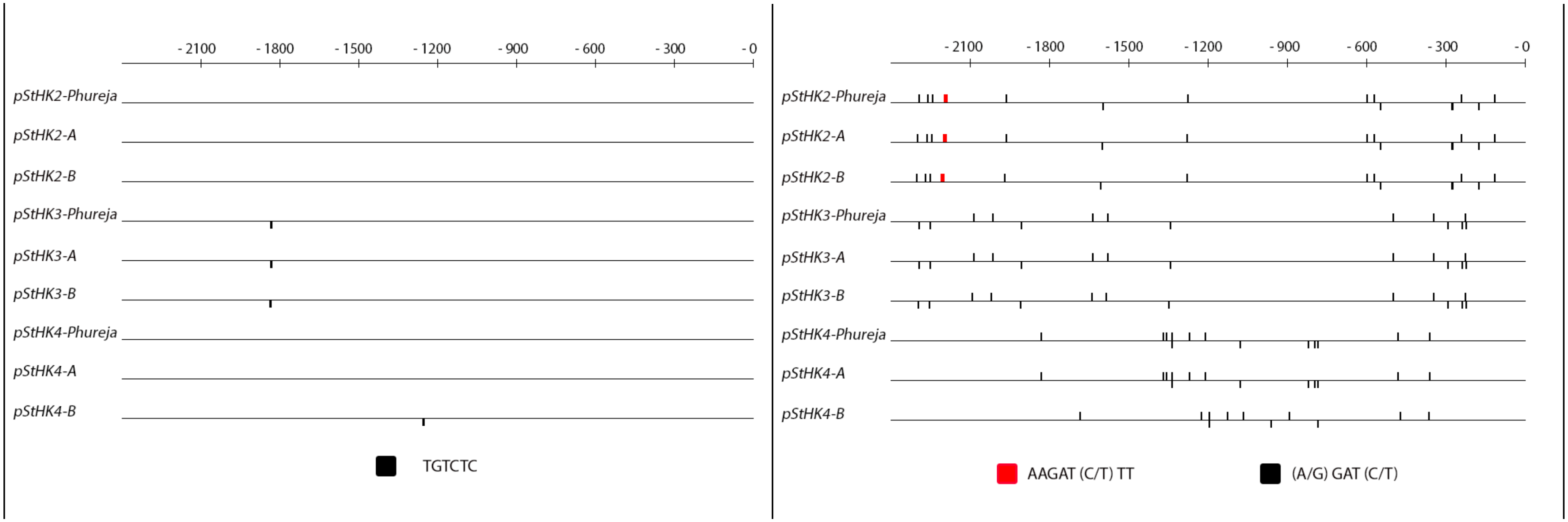

Figure S1. Comparison of hormone-responsive *cis*-element patterns in promoters of CK receptors from cv Phureja and cv Désirée. Positions upstream transcription start site AREs (on the left) and CRE (on the right) are denoted.
