# Supplementary material for: Hormonal Regulation and Crosstalk of Auxin/Cytokinin Signaling Pathways in Potatoes In Vitro and in Relation to Vegetation or Tuberization Stages
Source: Int J Mol Sci. 2021 Jul 30;22(15):8207. doi: 10.3390/ijms22158207 (PMC8347663; doi:10.3390/ijms22158207)
Supplement: Supplementary file 1 [file ijms-22-08207-s001.zip › Table S3.pdf]

Table S3. Quantitative gene expression in control potato plants grown on media with 1.5% or 5% sucrose.

Genes	Organs	1.5% Sucrose	5% Sucrose	*p*-value (t-test)
StTIR1a	Leaves	861.34	307.16	***0.037***
	stems	277.46	215.03	0.287
	Roots	303.00	172.92	*0.089*
StTIR1b	Leaves	92.90	32.89	***0.021***
	stems	56,73	43.19	0.330
	roots	61.77	35.59	0.126
StTIR1c	Leaves	39.54	6.23	0.116
	stems	38.63	29.08	0.268
	roots	14.46	11.46	0.361
StAFB4	Leaves	379.31	114.00	***0.039***
	stems	608.30	349.79	0.185
	roots	593.50	200.99	0.175
StAFB6	Leaves	435.55	212.64	0.167
	stems	318.72	168.31	0.143
	Roots	393.08	164.28	*0.053*
StIAA2	Leaves	3740.56	1037.88	***0.038***
	Stems	1765.62	933.62	0.147
	Roots	1658.50	480.82	***0.026***
StIAA3	leaves	2293.82	1176.20	0.202
	stems	2039.02	1486.37	0.172
	roots	1008.19	754.08	0.362
StIAA12	leaves	509.43	73.14	0.191
	stems	272.53	217.40	0.361
	roots	356.44	205.08	0.342
StIAA15	leaves	548.17	115.82	0.242
	stems	185.88	187.67	0.488
	roots	336.81	230.24	0.390
StARF2a	leaves	340.43	339.53	0.499
	stems	571.47	254.95	0.170
	Roots	354.56	131.31	***0.015***
StARF4	leaves	104.89	121.22	0.424
	stems	331.66	227.15	0.327
	roots	109.11	74.40	0.169
StARF5	leaves	19.04	19.57	0.490
	stems	94.02	151.34	0.347
	roots	203.14	93.97	0.284
StARF6	leaves	34.59	18.63	0.186
	stems	304.25	123.96	0.209
	roots	128.35	28.26	0.215
StARF8b	leaves	23.88	22.86	0.482
	Stems	157.00	98.06	***0.038***
	roots	37.76	25.16	0.326
StARF18	leaves	9.09	18.24	0.268
	stems	56.60	59.34	0.464
	roots	84.93	28.82	*0.095*

StARF19a	leaves	138.32	101.32	0.342
	stems	256.40	203.80	0.368
	roots	184.79	116.39	0.170
StHK2	leaves	57.15	73.52	0.119
	stems	102.44	83.94	0.153
	roots	138.06	68.24	***0.014***
StHK3	leaves	113.56	124.75	0.359
	stems	102.41	118.03	0.264
	roots	278.84	114.36	***0.028***
StHK4	leaves	84.03	41.11	***0.021***
	stems	346.82	482.21	0.321
	roots	281.09	271.69	0.477
StHP1a	leaves	482.32	282.97	0.305
	stems	737.62	272.93	0.226
	roots	474.41	749.75	0.251
StHP1b	leaves	138.57	107.64	0.402
	stems	172.57	61.74	0.229
	roots	154.37	142.38	0.466
StHP4a	leaves	97.61	140.61	0.254
	Stems	20.59	2.80	***0.010***
	roots	4.94	1.52	*0.077*
StRR4	leaves	920.94	323.44	0.195
	stems	805.54	463.32	0.177
	roots	672.75	838.59	*0.097*
StRR9a	leaves	710.09	92.38	0.203
	Stems	560.63	187.64	***0.018***
	roots	445.06	179.36	0.140
StRR9c	leaves	48.90	27.52	0.302
	stems	157.65	209.09	0.141
	roots	129.12	167.72	0.183
StRR9d	leaves	338.03	61.98	0.228
	stems	394.71	110.26	0.301
	roots	704.11	233.15	0.155
StRR1a	leaves	88.81	99.69	0.354
	stems	59.62	60.45	0.480
	roots	89.07	83.93	0.420
StRR1b	leaves	119.59	164.46	0.249
	stems	131.36	156.11	0.138
	roots	285.81	333.89	0.335
StRR11	leaves	19.11	11.05	0.284
	stems	50.64	27.25	0.102
	roots	63.29	25.60	***0.050***
StRR14	leaves	24.48	14.31	*0.063*
	stems	34.94	16.28	*0.053*
	Roots	30.45	12.39	***0.002***
StRR18a	leaves	68.84	42.36	***0.024***
	stems	160.93	300.85	***0.043***

	roots	214.17	125.03	***0.035***
StRR18b	leaves	63.56	71.37	0.346
	stems	87.51	60.07	0.173
	Roots	171.18	73.33	***0.021***

Relevant differences between 5% and 1.5% columns (fold changes > 2) are marked blue (down-regulation). Significant differences ($p < 0.05$) are marked in bold italics (right column), marginal differences ($p < 0.1$) are marked in italics.
