# Supplementary material for: Hormonal Regulation and Crosstalk of Auxin/Cytokinin Signaling Pathways in Potatoes In Vitro and in Relation to Vegetation or Tuberization Stages
Source: Int J Mol Sci. 2021 Jul 30;22(15):8207. doi: 10.3390/ijms22158207 (PMC8347663; doi:10.3390/ijms22158207)
Supplement: Supplementary file 1 [file ijms-22-08207-s001.zip › Table S4.pdf]

Table S4. A comparison of *cis* response elements (ARE, CRE) content in promoters of functional gene clades associated with auxin/cytokinin signaling with their transcriptomic responses to auxin (IAA) or cytokinin (BA) treatments of the whole 'Désirée' potato plants (1 μM, 1 h) in vitro.

**Gene Clade**	**Auxin Related[1]**		**CK Related[1]**	
	**ARE**	**DEG[2]**	**CRE[3]**	**DEG[2]**
StTIR1/AFB	0.667	1.043	0.667	1.104
StAux/IAA	1.750	2.835	1.000	1.259
StARF	0.167	0.986	1.167	1.148
StHK	0.333	0.863	1.000	1.105
StHPt	0.667	1.265	0.333	1.076
StRR-A	0.750	1.133	3.750	1.757
StRR-B	0.250	1105	1.500	1.609
Correlation coefficient[4]		0.93		0.87

[1]All data in the table are presented per gene averaged.

[2]Each number in DEG column is a normalized integral indicator which characterizes the averaged hormonal effect on the expression of a distinct gene clade. Here each gene expression value is a mean of seven DEG index determinations of the given gene expressed in the main potato organs regardless of the sucrose content in the growth medium.

[3]Each value represents normalized number of CREs in 300 bp-proximal promoter area.

[4]Extent of correlation (CC) between two columns of each pair was calculated by means of the Excel program.
